# Empyema or Suppurative Pleuritis

**Published:** 1879-08-20

**Authors:** F. M. Rushing

**Affiliations:** Alabama


### *A CASE OF EMPYEMA OR SUPPURATIVE PLEURITIS.*

BY F. M. RUSHING, M.D., OF ALABAMA.

On the 6th day of March, 1879, I was summoned to see a little boy aged nearly eight years. On my arrival the father informed me that he had been sick seven weeks, and the attending physician diagnosed the case pleuro-pneumonia. He was very anemic, with humid respiration and considerable dyspnea; pulse 150, and heart beating on right side with all the other symptoms indicating a collection of fluid in the left pleural cavity. I communicated to the father his true condition, and advised thoracentecis (I was not positive as to the nature of the accumulation). The father objected, and desired me to try some other method; I prescribed a purgative with a small quantity of mercury; a tonic composed of quinine, iron and capsicum, every three hours, and also sprt. nitre and digitalis as a diuretic. On the 9th and 11th I visited the case; he grew gradually worse; I had to prescribe opiates to procure any rest at all. I notified the father I could do nothing more unless he would allow me to operate.

On the 14th I was sent for, and when I arrived the father had left word for me to do whatever I thought best.

There being no place at which the accumulation seemed inclined to point, I punctured the skin between the 5th and 6th ribs, a little back of the median line, and thrust an ordinary trocar into the left pleural cavity. After about a quart of pus had passed out it became so thick it would not run through the canula. Not having a syringe with me to wash out the cavity, I did nothing more.

On the 20th I again punctured the chest at the same point, with the same instrument and drew out nearly half gallon of pus. I injected (through the canula, with a pump syringe), warm water and carbolic acid until the cavity was thoroughly cleansed out. I enlarged the opening with a probe-pointed bistoury, inserted an india rubber tube and advised the washing out of the cavity every morning with the carbolic acid and warm water.

On the 31st I was again summoned to see the little sufferer; the tube having come out and the opening nearly closed, I drew off over a quart of very fetid pus and again washed out the cavity with the carbolic solution; I then enlarged the opening, inserted a piece of lint and recommended a wash of the carbolic acid one morningand comp. tinct. iodine next, and to be kept up in this way.

On the 22d April I was summoned hurriedly to see my little patient, the messenger stating that he did not think he would live until I could get there. When I arrived I found that the opening had become clogged up; I removed the obstruction, enlarged the opening and drew off a quart of very fetid matter. I washed out the cavity with an acidulated solution of sulphurous acid, and the fetor entirely disappeared. The child was having hectic fever and was reduced so low I did not think he could live forty-eight hours. I ordered egg and brandy and gave the quinine and iron every two hours with an occasional dose morphine and as much sweet milk as could be taken. I ordered the sulphurous acid solution to be used every morning until the cavity was well cleansed out. The accumulation gradually diminished; the offensiveness of the discharge immediately disappeared, and by the 10th of May the opening had closed and the child was entirely well and hearty, and has remained so.

I give this report merely to strengthen the testimony heretofore given in favor of sulphurous acid as an antiseptic and in checking suppuration.

